# Full Characterization of Self-Compacting Concrete Containing Recycled Aggregates and Limestone

**DOI:** 10.3390/ma16175842

**Published:** 2023-08-26

**Authors:** Meriem Guessoum, Fouad Boukhelf, Fouzia Khadraoui

**Affiliations:** Builders Ecole d’Ingénieurs, Builders Lab, ComUE NU (Communauté Universitaire de Normandie Université), 1 Rue Pierre et Marie Curie, 14610 Epron, France; meriem.guessoum@builders-ingenieurs.fr (M.G.); fouzia.khadraoui-mehir@builders-ingenieurs.fr (F.K.)

**Keywords:** recycled concrete aggregates, limestone, self-compacting concrete, fresh properties, durability

## Abstract

This work deals with the study of self-compacting concretes (SCCs) containing recycled aggregates (RAs) recovered from demolition waste and limestone filler as a partial replacement for natural aggregates (NAs) and cement, respectively. Four mix designs were developed and characterized in both the fresh and hardened states. In the fresh state, the properties studied included slump, sieve stability, and t_500_ viscosity. In the hardened state, the properties studied were compressive strength and porosity at 15 h and 28 days, thermogravimetric analysis, and durability tests involving freeze–thaw cycles and accelerated carbonation. The results indicate the RAs lead to a decrease in slump flow. However, the substitution rate of aggregate replacement does not affect the compressive strength. This can be attributed to the optimized mix design, resulting in all SCC mixtures achieving the same compressive strength class of 30–35 MPa. As for the durability tests, the incorporation of recycled aggregates modifies the behavior of the concrete during freeze–thaw cycles. Throughout the 300 freeze–thaw cycles, all concrete mixtures exhibited a mass loss accompanied by a slight strain increase, but the materials remained visually intact. Additionally, the carbonation depth is strongly influenced by the rate of aggregate replacement due to changes in the microstructure, particularly in porosity.

## 1. Introduction

Concrete is the most widely used construction material globally, with an annual production exceeding 10 billion m^3^ [1]. In terms of regional consumption, Europe stands out with an annual consumption of concrete surpassing 620 million m^3^. This highlights the significant demand for concrete in construction activities throughout the continent. Specifically, in France, the production of concrete amounts to over 21 million tons per year. This substantial production volume contributes to the country’s construction industry, generating a considerable sales revenue of 19 billion euros, according to the French Federation of the Concrete Industry [2].

However, despite its advantageous qualities in terms of mechanical strength, durability, and cost-effectiveness, concrete has significant drawbacks related to its composition and manufacturing processes. The concrete industry is known for its high energy consumption and adverse environmental impact, particularly in terms of greenhouse gas (GHG) emissions. It is estimated that the production of concrete contributes to 5.2% of global GHG emissions. One of the main contributors to these emissions is cement production, which alone accounts for 52% of the total emissions associated with concrete. In France, for instance, the production of one ton of cement emits an average of 0.62 tons of CO_2_eq [3].

Another concerning aspect is the extensive use of concrete in construction, which has additional negative consequences for the environment. One such consequence is the depletion of non-renewable natural resources, particularly sand and gravel, which are essential components of concrete production [4]. The extraction of these resources can lead to habitat destruction, erosion of riverbanks, and disruption of ecosystems. In concrete, aggregates are the most important part, accounting for around 3/4 of the total volume of the concrete mix [5]. The demand for aggregates tends to increase with the growing demand for concrete. In this context, the use of recycled aggregates in the manufacture of concrete is a method of circular economy that meets the needs of sustainable construction while preserving the environment [6,7].

In addition to its contribution to global warming, the blatant use of concrete leads to a shortage of non-biodegradable natural resources, namely sand and gravel, which are essential for its manufacture [4]. Furthermore, the demolition of concrete at the end of its life generates considerable quantities of waste occupying large surfaces. This waste represents about 36% of the total waste produced on earth [8]. The environmental impact of the concrete industry extends beyond carbon emissions and resource depletion. The indiscriminate use of concrete results in significant amounts of waste when buildings or infrastructure reach the end of their lifespan. In the United States, concrete waste has significantly increased from 50 million tons in 1980 to 548 million tons in 2015 [9]. In China, this amount exceeds 1500 million tons [10]. In Europe, approximately 850 million tons of concrete waste is produced each year, accounting for about 31% of the total waste production in the European Union [11] and about 36% of the total waste produced on earth [8].

Nowadays, the use of recycled aggregate in the manufacture of new concrete is becoming increasingly widely practiced. Recycled aggregate is obtained by processing concrete waste, demolishing structures, or reclaiming materials from other sources. The main advantage of using recycled aggregate is that it reduces the amount of construction waste sent to waste disposal sites, thus helping to preserve the environment. In addition, its use reduces the demand for natural materials, such as virgin aggregates extracted from quarries, thus helping to preserve natural resources. Although recycled aggregate may have slightly different particle sizes and properties compared to natural aggregates, it can be adapted and used effectively in the manufacture of concrete. Studies and research have been carried out to determine the appropriate proportions of recycled aggregate to use in each type of concrete, considering the required mechanical properties [12,13]. The use of recycled aggregate in new concrete does not compromise the quality and performance of the final material. In fact, it has been demonstrated that concretes containing recycled aggregate can perform comparably to, or even better than, traditional concretes with natural aggregates. Moreover, the use of these recycled materials can contribute to obtaining environmental certifications for construction projects by reducing the overall carbon footprint [14]. In order to address these problems, the use of recycled aggregate and alternative binders in concrete mix designs is necessary and constitutes an unavoidable solution [15]. The path towards the production of concrete that is more respectful of the environment and less polluting for the planet will be confronted with several important challenges, including rheological and mechanical performances, durability, and adaptability to the intended application. However, the use of recycled aggregates in the development of self-compacting concrete (SCC) is justified and technically feasible [16,17,18], although it is imperative to adjust the mix design each time due to the irregularity of the intrinsic characteristics of recycled aggregates. For this replacement to be effective, it is necessary to remedy the adverse effect of old mortar on SCC containing recycled aggregates. Potential aggregate treatment methods have been investigated to promote the maximum use of recycled aggregates [19]. Nevertheless, it is necessary to monitor changes in mechanical strength and durability over time. To this end, indirect estimation of these properties through non-destructive testing is now possible [18].

Several current studies aim to find the appropriate mix design of low-carbon self-compacting concretes based on recycled gravel, obtained by adding a superplasticizer whose excess can lead to segregation and by incorporating mineralogical additives and recycled aggregate at relatively high rates [20], without the latter adversely affecting the mechanical and rheological performance of the concrete [21]. Therefore, extensive research and testing are required to optimize the mix design of low-carbon self-compacting concretes based on recycled aggregate [22]. This includes the careful addition of a superplasticizer to prevent segregation and the incorporation of mineral additives and recycled aggregate at high rates without compromising the mechanical and rheological properties of the concrete [23,24,25].

In fact, limestone fillers are widely used in the production of SCC [14]. Limestone fillers, also known as fine limestone powders, are fine materials that can partially replace cement or traditional fillers (such as silica fillers) in the composition of SCC. Limestone fillers offer several advantages in SCC. Firstly, they help to improve the workability of the mix [26], enabling better particle distribution and filling of voids [27]. This facilitates concrete placement without the need for vibration, reducing construction time and, thus, labor costs. Limestone fillers also have a beneficial effect on the mechanical properties of concrete [28].

To address the environmental issues associated with concrete production, the use of recycled gravel and alternative binders in concrete mix designs is indeed a necessary and viable solution. This approach helps to reduce the need for natural aggregates, as well as the depletion of natural resources and the carbon footprint. However, implementing these changes in concrete production does come with several significant challenges, namely rheological and mechanical performances, durability, and adaptability to the intended application.

## 2. Materials and Methods

**Figure 1:**
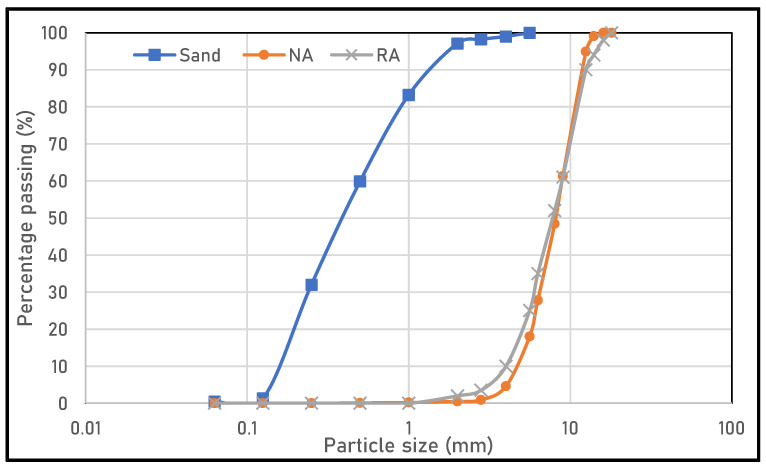
Particle size analysis curve.

Experiments were carried out to investigate the partial or total replacement effect of aggregates by those resulting from the demolition of old buildings. The use of recycled aggregates would affect fresh and hardened properties of SCC. For all mix designs, cement (CEM I-52.5 R) provided by AALBORG^®^ (Rochefort, France) was replaced with limestone filler produced by Omya^®^ (Noisy le Roi—Yvelines, France) with a mass replacement ratio of 35%. Sand and coarse natural aggregates are classified as 0/4 and 4/12.5, respectively. Recycled aggregates are classified as 4/14, and their property classification is type 1 according to EN 206-1 [29]. Figure 1 presents the particle size analysis curve for the aggregates used. It can be seen that recycled aggregates are coarser than natural ones but contain fine material derived from the dust of the old cementitious matrix of the old concrete. Table 1 provides the amounts in kg/m^3^ of the different constituents of the SCC. In fact, in the case of self-compacting concrete, the volume of binder paste should be around 40% of the total volume, to the detriment of the volume of aggregates.

**Figure 2:**
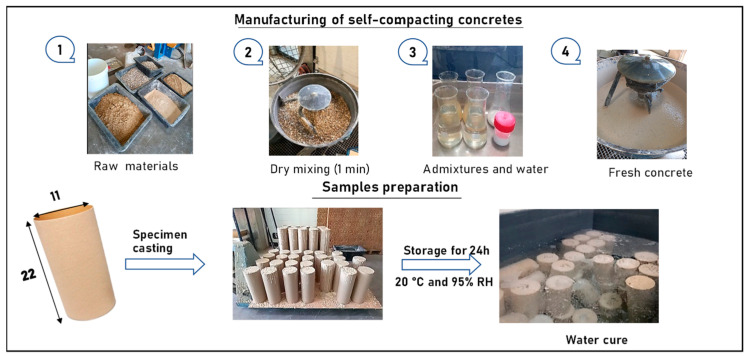
Specimen preparation.

Following the steps presented in Figure 2, The raw materials were prepared and weighed before dry mixing for 1 min. After that, water and admixture were added, respectively. Indeed, the target concrete class was C30/37 with a consistency of SF2. Four concrete mix designs were studied: a reference concrete with natural aggregates (NAs) and three concretes incorporating recycled aggregates (RAs). The substitution rates of NA by RA are 20, 50, and 100%. These are noted 0%RA, 20%RA, 50%RA, and 100%RA, respectively. The aggregates were stored under ambient conditions (Temperature T = 20 °C and relative humidity RH = 50%). All concretes were manufactured at a temperature of 23 °C. The 11 cm × 22 cm cylindrical specimens were demolded 24 h after production and stored at T = 20 °C and RH = 95%. A total of 28 specimens for each mix design were made to study the property evolution at a hardened state in the laboratory for 15 h and 28 d. In order to assess the RA effects, the different concrete compositions were characterized in terms of slump flow, segregation strength, and t_500_ according to the European standard EN 12350-8 [30], critical sieve stability according to the European standard EN 12350-11 [31], compressive strength according to the French standard NF EN 12390-3 [32], thermogravimetric analysis (TGA) and its derived factor (dTG), and water absorption and porosity according to AFPC-AFREM [33].

From the durability test point of view, the extreme freeze–thaw test was carried out on 3 test specimens measuring 10 cm × 10 cm × 40 cm for each mix design according to the European standard NF P 18-424 [34]. The test specimens were placed in a freezing chamber, allowing 300 cycles of temperature variation to be reproduced at a freezing rate of 6 °C/h until −18 °C was reached. The freeze period was maintained for 30 min, followed by a programmed thaw period of 30 min at 9 °C with a 45 min rise time. Test specimens were recovered every 30 cycles after thawing, wiped dry, and stored at room temperature for 90 min. Two measurements were carried out, the first measuring specimen strain and the second mass loss. In addition, concrete carbonation depth was measured on 7 cm × 7 cm × 28 cm prismatic specimens placed in a carbon incubator with a regulated CO_2_ concentration of 3%, a temperature of 23 °C and a relative humidity of 60%, in accordance with standard XP P18-458 [35]. After 7, 28, and 70 days of carbonation, the prisms melted in the transverse direction. Immediately after splitting, the surface was wiped clean, and phenolphthalein solution was sprayed on, allowing the pH of the surface to be measured. Phenolphthalein is colorless at a pH below 8.2, corresponding to a change in microstructure, particularly portlandite phase, which becomes carbonated and generates a calcite phase, and deep pink at a pH above 9.9. In general, 12 depth measurements, i.e., the colorless part, were taken for each sample.

## 3. Results and Discussion

### 3.1. Fresh State

**Figure 3:**
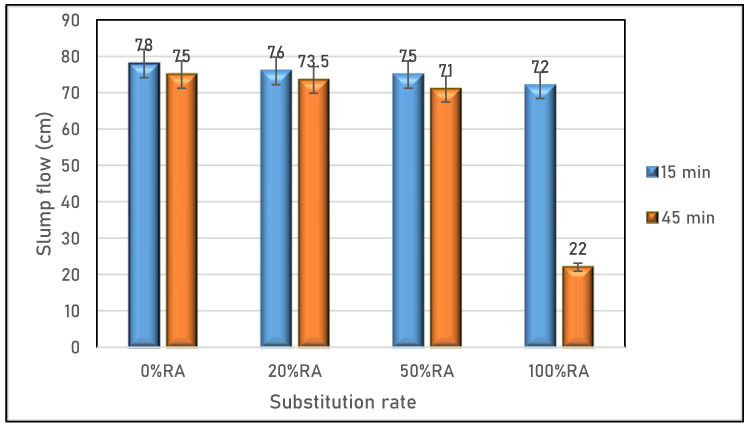
Slump flow of SCC studied.

Figure 3 illustrates the slump flow results 15 and 45 min after mixing for the studied SCC. The findings indicate that the variation in workability at 15 min after mixing was not significant for the SCC based on RA compared to the reference SCC (0%RA). This conservation of workability can be attributed to the presence of superplasticizers in the different SCC mix designs. However, a significant change in workability occurs at 45 min after mixing was observed for the SCC completely formulated with RA (100%RA). The use of RA changed the rheological properties and extended the workability maintenance period. Whereas, in the case of 100%RA, the SCC lost its fluidity. This change can be explained by the presence of fines from old concrete, which increases the water demand and adversely affects workability 45 min after mixing.

**Figure 4:**
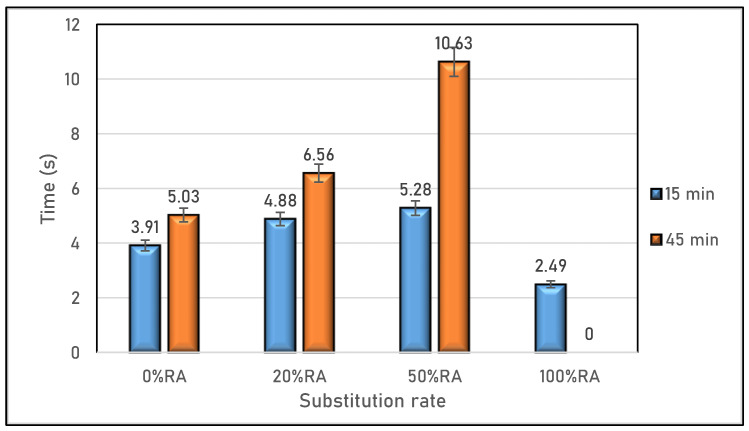
Viscosity of SCC studied.

As for viscosity, measured by the time it takes for the slump flow to reach a diameter of 500 mm (t500) and depicted in Figure 4, an increase in the slump flow time was observed with the rise in RA replacement rate. This increase is a consequence of RA being less mobile due to the decrease in paste content [36]. In particular, the 100%RA mix design loses its workability and ceases to flow. This can be attributed to the presence of excess fine material in the RA, leading to a higher water demand [15]. This higher water demand is associated with the increased water absorption observed when the RA replacement ratio is raised [36,37].

**Figure 5:**
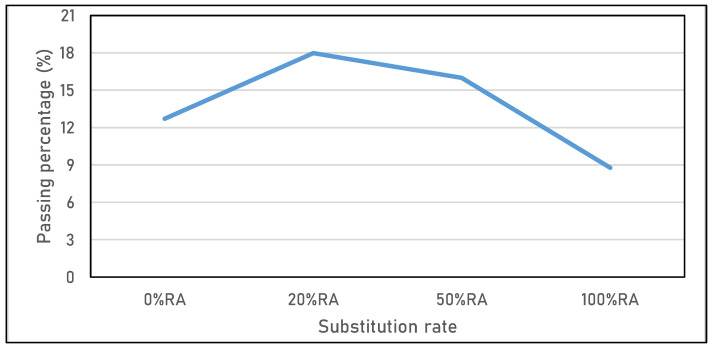
Sieve stability of SCC studied.

Figure 5 shows an assessment of the segregation resistance determined by measuring the amount of laitance passed through a 5 mm sieve. The results obtained by the sieve stability test qualify the 0%RA and 100%RA as stable concretes, the percentage of laitance being lower than 15%, which expresses a good segregation stability for these two concretes. Contrary to the 20%RA and 50%RA, which represent critical stability, expressed a laitance exceeding 15%. This can be attributed to the granular discontinuity between RA and NA used in these two mix designs. In agreement with the literature, the segregation tended to decrease with the increased substitution rate. This is due to the high water absorption capacity of RA with fine material [36,37].

### 3.2. Hardened State

**Figure 6:**
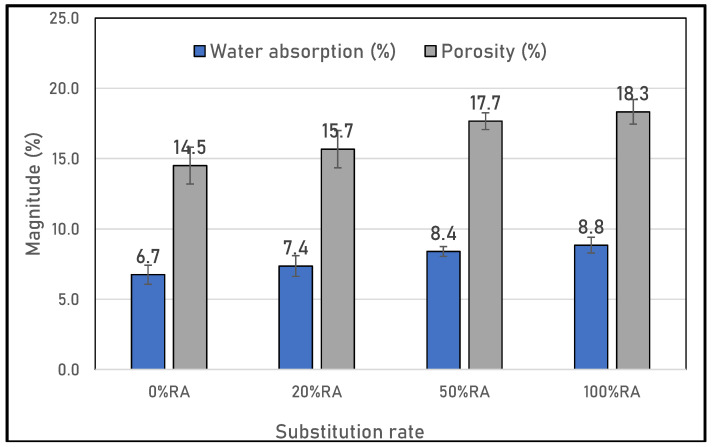
Porosity and water absorption of SCC studied.

The water-accessible porosity and water absorption results at 28 days are presented in Figure 6. An increase in porosity and water absorption with the increase of the RA substitution rate was observed. Indeed, the porous structure of the SCC based on RA was related to the presence of an interfacial transition zone (ITZ) between the old and new mortars, according to what is found in the literature [38]. In general, concrete consists of a blend of aggregate, cement, and water, with each of these components contributing to the strength and durability of the building material. However, the presence of distinct elements and their interactions results in the formation of an interfacial transition zone (ITZ) [39]. This ITZ represents the most vulnerable connection between cement mortar and coarse aggregates. It occupies a range of 20–40% of the overall volume of cementitious material, typically measuring around 40 μm in thickness [40]. The ITZ has a significant impact on the mechanical and long-lasting qualities of solidified concrete. Often, concrete failures occur at the ITZ due to its limited capacity for stress transmission [41].

In standard concrete, a single ITZ exists, positioned between mortar and natural coarse aggregate. However, when incorporating recycled aggregate sourced from construction and demolition waste, a three-phase composite emerged. This composite includes aged natural aggregate, the associated cement mortar, and the previous ITZs between these materials. Consequently, the integration of recycled aggregate introduces at least two distinct types of ITZs into the concrete matrix [42]. These new ITZs can be further categorized into two groups: the ITZ between old and new mortar, and the ITZ between the aged aggregate surface and new mortar, as well as the pre-existing ITZ between the original aggregate and its attached aged mortar.

In addition, due to the consistency required in the fresh state, the 100%RA contained more water than the other mix designs due to the high absorption of recycled aggregate, as shown in Table 1 [15]. This can lead to the formation of voids in the cementitious matrix and inefficient packing of the binder particles with the aggregates [43].

**Figure 7:**
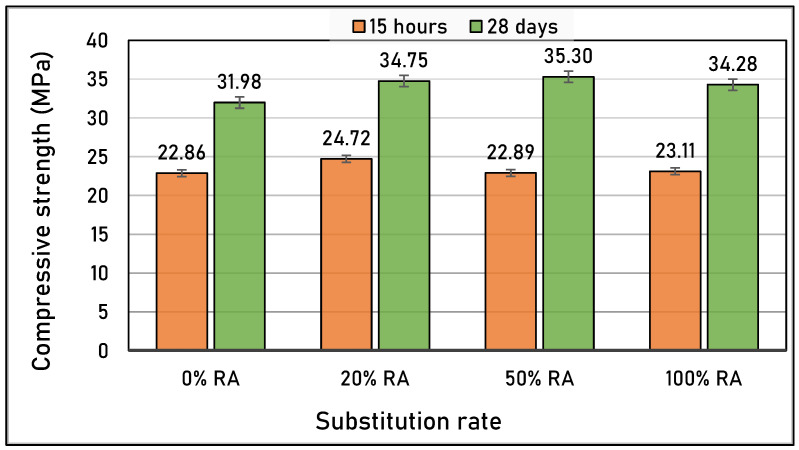
Compressive strength at 15 h and 28 d of studied concretes.

Figure 7 presents the compressive strength values at 15 h and 28 d of curing. In general, concretes reach a strength higher than 22 MPa after 15 h of curing. This high strength is the result of the use of 52.5 R cement, which has a short-term rapid hardening, in addition to the use of limestone fillers that close the pores. It was also observed that all the SCCs made with RA had better compressive strength than the natural SCC (0%RA), even at an early age. Indeed, the incorporation of RA, even with high percentages in the SCCs, improved their mechanical performance. The SCC mix designs with RA had a higher compressive than NA because the recycled aggregate’s physical and mechanical characteristics improved upon interlocking with the cementitious matrix [44]. In fact, the RA used was type 1, which comes from old concrete and contains no plastic, gypsum, masonry, or glass waste.

**Figure 8:**
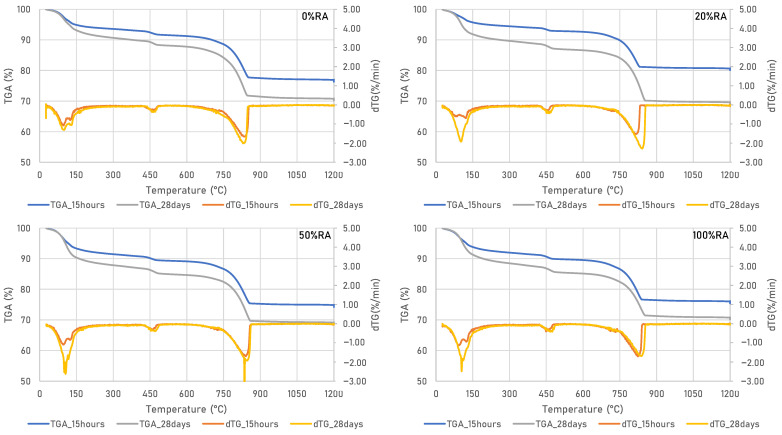
TGA and dTG at 15 h and 28 d of SCC studied.

In order to discuss the mechanical strength results, thermogravimetric size analysis was carried out on all concrete at 15 h and 28 d of curing. The results shown in Figure 8 indicate the presence of three phases. The first is attributed to the departure of (absorbed) water from the constitution of certain hydrates such as C-S-H and ettringite AFt at 100 to 130 °C. The second is linked to the dehydration of the water chemically bound with the portlandite (Ca(OH)_2_) at a temperature of between 400 and 480 °C, and the last relates to the decarbonation of the calcite phase (CaCO_3_) at between 750 and 850 °C [45]. Furthermore, a variation in hydrate quantities was observed for samples tested at 15 h and 28 d. This variation is explained by the kinetics of the hydration process of cement and limestone fillers. These concretes contain less of the portlandite phase and more calcite as a result of the addition of limestone fillers, which are very rich in CaCO_3_ [46]. The findings show that RA has no effect on the hydration process in terms of CSH gel formed, ettringite, portlandite, and calcite. This is due to the quality of the recycled aggregates, which are type 1 according to EN 206-1 [29] and contain only old concretes.

### 3.3. Durability

#### 3.3.1. Freeze–Thaw Results

**Figure 9:**
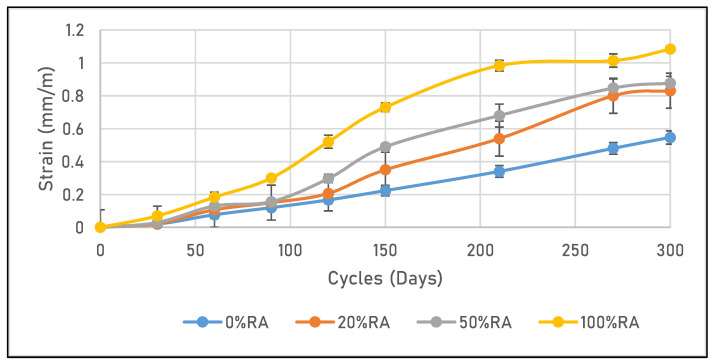
Strain due to the freeze–thaw cycles of tested concretes.

**Figure 10:**
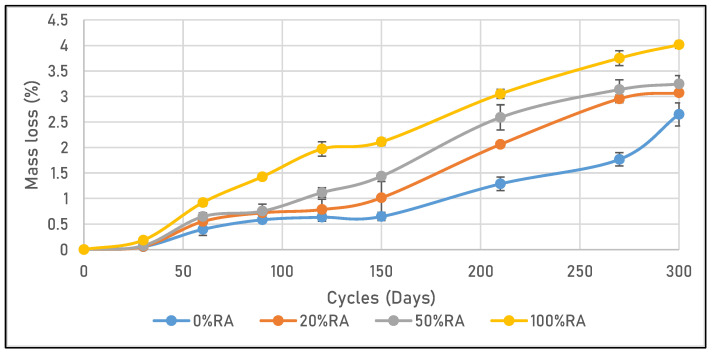
Mass loss due to the freeze–thaw of tested concretes.

Figure 9 shows a quasi-linear increase in strain with freeze–thaw cycles for the 0%RA material and a non-linear increase for other mix designs. In addition, the maximum strain was 0.55, 0.8, 0.85, and 1.2 mm/m for 0%GR, 20%GR, 50%GR, and 100%GR, respectively. From the mass loss point of view, the expansionary behavior of the concrete samples in response to freeze–thaw cycles led to a weakening and cracking of the surface material, resulting in a mass loss (cf. Figure 10). All these results are in accordance with those for porosity. In fact, freeze–thaw behavior was improved by replacing part of the cement with limestone fillers, as demonstrated by Zeng et al. [46].

#### 3.3.2. Accelerated Carbonation

**Figure 11:**
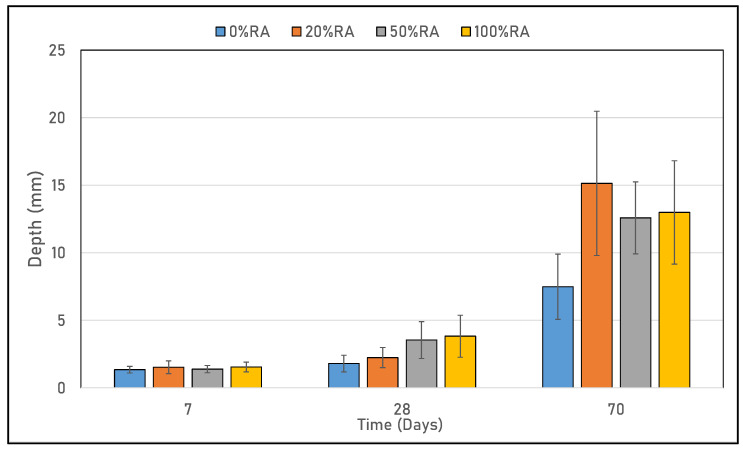
Carbonation depth of tested concretes.

The accelerated carbonation results in Figure 11 show that the rate of CO_2_ penetration was proportional to the exposition time [47]. This is explained by the synthesis process between the cementitious matrix and the absorbed CO_2_, which reduces the pH of the pore solution [48], resulting in the formation of the calcite phase (CaCO_3_) to the detriment of the calcium silicate hydrate (CSH), the portlandite phase (Ca(OH)_2_), and calcium oxide (CaO), as shown by [49]. Moreover, all mix designs had the same CO_2_ uptake at 7 days of exposition. After that, the carbonation depth increased with the replacement rate of the recycled aggregates. In general, the accelerated carbonation results were in accordance with those for porosity related to the ITZ. Indeed, CO_2_ penetrates through the concrete’s pore network and reacts with the concrete’s old and new cementitious matrix [50]. Furthermore, the carbonation rate of the concretes studied was calculated in accordance with standard FD P 18-480 [51] and showed a rate of 1.8 mm/day^0.5^ for all SCCs based on RA, (20%RA, 50%RA, and 100%RA) and 0.96 mm/day^0.5^ for the reference concrete (0%RA), which, for our mix designs, can be used in a carbonation exposure class of XC4 according to EN 206-1 [29]. This change may be related to the fact that RA containing old cementitious matrix in its interface comes from the already carbonated concrete of old building materials [52].

## 4. Conclusions

This study is part of an environmental transition process aimed at reducing the carbon footprint associated with construction materials and addressing the issue of non-biodegradable waste occupying vast landfill areas. The focus of this research was on investigating the properties of self-compacting concretes containing 35% limestone and recycled aggregates in both fresh and hardened states, including slump flow, t_500_ viscosity, sieve stability, porosity and water absorption, mechanical strength, thermogravimetric analysis, freeze–thaw, and accelerated carbonation. The results obtained are encouraging and align with the specified requirements and intended applications. The findings are outlined as follows:

- The use of recycled aggregates reduces the time required to maintain consistency due to the fine particles present in the aggregates and their significant water absorption.
- The sieve stability of the 20%RA and 50%RA self-compacting concretes was greatly modified by the granular discontinuity, in contrast to homogeneous aggregate mix designs such as 0%RA and 100%RA.
- The recycled aggregates do not affect either the mechanical compressive strength or the binder hydration, as demonstrated by TG analyses.
- The self-compacting concretes tested exhibit good freeze–thaw resistance, and the difference between them is strongly related to the porosity. The 0%RA presents a quasi-linear behavior, and this increases with the higher replacement rate by 45%, 54%, and 110% for 20%RA, 50%RA, and 100%RA, respectively.
- The developed self-compacting concretes have a carbonation rate of 0.96 mm/day^0.5^ for the 0%RA and about 1.8 mm/day^0.5^ for the other mix designs.

The comprehensive experimental characterization campaign presented in this work and the resulting findings constitute valuable additions to the database on self-compacting concretes. Another study will be carried out on the application of these materials in a demonstrator building.

## Figures and Tables

**Table 1:** Mix design of SCC studied (kg/m^3^).

	0%RA	20%RA	50%RA	100%RA
Sand 0/4	786	786	786	786
Natural aggregate 4/12.5	726	580.8	361	-
Recycled aggregate 4/14	-	145.2	361	726
Blanc CEM I-52.5 R	320	320	320	320
Limestone	160	160	160	160
Efficient water	161.81	161.81	161.81	179
Total water	168	168	168	185.19
Superplasticizer	4.8	4.8	4.8	4.8
Set accelerator	3.80	3.80	3.80	3.80
Water-repellent	0.78	0.78	0.78	0.78

## Data Availability

The experimental and computational data presented in the present paper is available from the corresponding author upon request.
